# Physiological and Molecular Characterization of Biosurfactant Producing Endophytic Fungi* Xylaria regalis* from the Cones of* Thuja plicata* as a Potent Plant Growth Promoter with Its Potential Application

**DOI:** 10.1155/2018/7362148

**Published:** 2018-05-13

**Authors:** Mohd Adnan, Eyad Alshammari, Syed Amir Ashraf, Kartik Patel, Kishan Lad, Mitesh Patel

**Affiliations:** ^1^Department of Clinical Laboratory Sciences, College of Applied Medical Sciences, University of Ha'il, P.O. Box 2440, Ha'il, Saudi Arabia; ^2^Department of Clinical Nutrition, College of Applied Medical Sciences, University of Ha'il, P.O. Box 2440, Ha'il, Saudi Arabia; ^3^Bapalal Vaidhya Botanical Research Centre, Department of Biosciences, Veer Narmad South Gujarat University, Surat, Gujarat, India

## Abstract

Currently, there is an absolute concern for all nations in agricultural productivity to meet growing demands of human population. In recent time, biosurfactants produced by diverse group of microorganisms are used to achieve such demands as it is known for its ecofriendly use in elimination of plant pathogens and for increasing the bioavailability of nutrients for plants. Endophytic fungi are the important source of secondary metabolites and novel bioactive compounds for different biological applications. In the present study, endophytic fungi* Xylaria regalis (X. regalis)* recovered from the cones of* Thuja plicata* was evaluated for its biosurfactant producing ability and plant growth-promoting abilities through various screening methods and also via its antagonistic activity against phytopathogens like* Fusarium oxysporum and Aspergillus niger*. In addition,* X. regalis* was also tested* in vivo* for a various range of growth parameters in chilli under greenhouse conditions. Significant increase in shoot and root length, dry matter production of shoot and root, chlorophyll, nitrogen, and phosphorus contents of chilli seedlings was found, which reveals its ability to improve the growth of crop plants. Hence, this study suggests the possibility of biosurfactant producing endophytic fungi* X. regalis* as a source of novel green biosurfactant for sustainable agriculture to achieve growing demands.

## 1. Introduction

Due to various environmental changes, agricultural field confront numerous sorts of challenges like polluting influences in soil, low water ingestion capacity, pathogen attack, low plant to plant growth-promoting microorganism interactions, and so on. To defeat these sorts of challenges, there is a need of various ecofriendly biological amendments like biosurfactants. Large number of microorganisms produced low molecular weight surface active molecules which are known as “biosurfactants.” They possess high surface activity with high specificity and are derived from renewable resources, effective under extreme conditions, and non-toxic in nature, when compared to synthetic ones [1–3]. Biosurfactants have wide range of application in different fields like petroleum, medicine, food, pharmaceuticals, and cosmetics [4–8]. Recently, use of biosurfactants for agro-chemical formulations and crop protection is considered to be the most intensive area of research in agriculture.

In nature, plants have an intrinsic relationship with a broad range of microbes that colonize the phyllosphere (epiphytic bacteria), the rhizosphere (rhizobacteria), and plant tissue (endophytes) [9]. Endophytic fungi are those microorganisms, which are associated with plants and inhabit them for at least a certain period of time without causing any kind of adverse effects. Endophytic fungi can act as growth promoters [10], defenders against predators [11], and competitors of microbial pathogens [12]. This unique association between fungi and plant can result in enhancement of plant growth, tissue differentiation, and response against biotic and abiotic stresses without causing any diseases symptoms [13, 14]. However, recent studies have shown that endophytic fungi enhance vegetative growth in many grass species and increased plant fitness via secretion of plant growth-promoting secondary metabolites like gibberellins, auxin, cytokinin, and so forth [15–17]. Moreover, they are also recognized as potential source of many novel bioactive compounds for their exploitation in agriculture and horticulture [18, 19].

Endophytic fungi might produce variety of biosurfactant molecules which may help in plant growth promotion, directly or indirectly. However, there are only very few reports regarding the production of biosurfactants by endophytic fungi [20]. Relationship between biosurfactant producing endophytic fungi with their host plants is just in the beginning phase and is not well understood. In spite of ever increasing information about the application of biosurfactant in various fields, their functional application in plant growth promotion is limited especially in the field of agriculture. Therefore, this work aimed to isolate endophytic fungi from* Thuja plicata* (Donn ex. D. Don.) cones with molecular characterization and screening for their biosurfactant producing ability and plant growth-promoting activities.

## 2. Materials and Methods

### 2.1. Sample Collection and Isolation of Endophytic Fungi

*Thuja plicata* (Donn ex. D. Don.) cones were collected from the Bapalal Vaidhya Botanical Garden, Gujarat, India (21°09′08.97′′ N 72°46′57.63′′ E). Cones were placed in a sterile polythene bags and processed within 24 hours of collection. All the collected cone samples were washed with running tap water to remove debris and cut into small pieces. Pieces were surface sterilized by 70% ethanol for 10 seconds, followed by 4% sodium hypochlorite for 1 minute, and finally rinsed with sterile distilled water for 1 minute. Excess amount of moisture was removed by blotting. The cone tissue samples were placed in Petri dish containing potato dextrose agar (PDA) medium supplemented with chloramphenicol (100 mg/l). Plates were incubated at 28°C for one week and monitored every day for the growth of endophytic fungi. Pure culture of endophytic fungi was obtained by repeated streaking and stored at 4°C for further study.

### 2.2. Identification through DNA Extraction, PCR Amplification, and Sequencing

Genomic DNA was extracted from the fungal mycelium, directly collected from culture plates following the method of Płaza et al. [21]. Quantification of DNA was done according to the method described by Sambrook et al. [22]. 10 *μ*L of extracted DNA was dissolved in 30 *μ*l of Tris buffer (pH 8) and OD was taken at 260 and 280 nm (PowerWave HT Microplate Spectrophotometer, BioTek®). Quality was assessed by taking the (OD at 260 nm)/(OD at 280 nm). Amplification of Internal transcribed spacer gene (ITS) was carried out by using a pair of primer ITS1 and ITS4 with 1x final concentration of ReadyMix™ Taq PCR reaction mix (Sigma-Aldrich®, India) and template DNA (50 ng/*μ*l). The reaction was carried out in Thermal cycler (Applied Biosystems Veriti®). ITS1F 5′-TCCGTAGGTGAACCTGCGG-3′ was used as a forward and ITS4R 5′-TCCTCCGCTTATTGATATGC-3′ was used as a reverse primer. PCR reaction mixture contained 1x reaction mixture (10 *μ*L), forward primer (1 *μ*L), reverse primer (1 *μ*L), genomic DNA template (2 *μ*L), and nuclease free water (6 *μ*L). PCR program was adjusted as follows: initialization at 95°C for 3 min, 35 cycles of denaturation at 95°C for 30 sec, annealing at 55°C for 30 sec, and extension at 72°C for 1 min, followed by final elongation step at 72°C for 10 min with hold at −4°C for ∞ time. Amplified PCR products were detected on agarose gel (1%) by electrophoresis staining with ethidium bromide and visualizing under UV light. Purification of amplified PCR product was done using GenElute™ PCR Clean-up kit (Sigma-Aldrich, India). Purified PCR product was sequenced by Eurrofins Genomics India Pvt., Ltd., Bangalore. Sequence analysis was done using sequencing analysis software BioEdit 7.2.5 and subjected to sequence match analysis using Basic Local Alignment Search Tool (BLAST) on NCBI. Final edited sequences were submitted to GenBank database of NCBI.

### 2.3. Phylogenetic Analysis

ITS gene sequences of different* Xylaria* species were downloaded from GenBank in FASTA format. Pairwise alignment and multiple sequence alignment (MSA) were carried out by using Clustal-W embedded in Molecular Evolutionary Genetics Analysis (MEGA 7.0). All positions containing gaps and missing data were eliminated. Phylogenetic analysis was performed using maximum parsimony (MP) and maximum likelihood (ML) approach in MEGA 7.0. The MP tree was obtained using the Subtree-Pruning-Regrafting (SPR) in which the initial trees were obtained with heuristic searches of 1,000 replicates of random stepwise sequence addition. Phylogenetic analysis consisted of bootstrap resampling method of Felsensten [23]. The bootstrap consensus tree inferred from 1000 replicates is taken to represent the evolutionary history of the taxa analyzed. Branches corresponding to partitions reproduced in less than 50% bootstrap replicates were collapsed. Maximum likelihood approach was based on the Tamura-Nei model in MEGA 7.0, followed by 1000 replicates.

### 2.4. Screening of Endophytic Fungi for Biosurfactants Production

#### 2.4.1. Emulsification Capacity Assay

The emulsification index (% EI24) was determined according to Cooper and Goldenberg [24]. Isolated endophytic fungi were inoculated in to liquid media containing MgSO_4_ (0.5 g/L), Na_2_HPO_4_ (3.0 g/L), KH_2_PO_4_ (1.0 g/L), and yeast extract (1.3 g/L). After autoclaving, 0.5 g/L of olive oil was added in order to induce biosurfactant production [25]. Fungi were cultivated up to 5 days at 28°C and 140 rpm. At the end of incubation, culture was filtered and supernatant was used for emulsification capacity assay. Equal volume of culture supernatant and different surfactants (kerosene/aromatic/aliphatic/different oils) were mixed in a glass test tube with vortexing for 2 minutes and left to stand for 24 hours. % EI24 was calculated by using the following equation. A higher emulsification index indicates a higher emulsification activity of the tested surfactant:

(1) $$\begin{array}{c} \%\;\;EI24=\frac{\text{Height of emulsion formed}}{\text{Total height of the solution}}\times 100. \end{array}$$

#### 2.4.2. Oil Spreading Assay

The oil spreading assay was carried out as described by Youssef et al. [26]. 20 *μ*l of crude oil was placed on the surface of 50 *μ*l of distilled water in to a Petri dish. 10 *μ*L of culture supernatant from endophytic fungal isolate was put on the surface of the crude oil. The diameter of the clear halo zone was visualized under visible light and measured. Distilled water was used as negative control and sodium dodecyl sulfate (SDS) was used as a positive control.

#### 2.4.3. Drop Collapse Assay

The drop collapse test was performed according to Plaza et al. [27]. In this method, 5 *μ*l of crude oil was added to 96-well microtitre plate. Plate was equilibrated for 1 hour at room temperature and 5 *μ*l of culture supernatant from endophytic fungal isolate was added to the surface of the oil. Shape of drop on the oil surface was observed after 1 minute. If culture supernatant makes the drop collapse, this indicated as a positive result but if the drop remained beaded, the result was scored as negative. Distilled water was used as negative control and SDS was used as a positive control.

### 2.5. Blue Agar Plate (BAP) Method

Minimal agar medium with glucose (2%), cetyl trimethyl ammonium bromide (CTAB, 0.4 mg/mL), and methylene blue (0.2 mg/mL) were used to detect extracellular biosurfactants [28]. 25 *μ*l of culture supernatant was added to each well and incubated for 48–72 hours at 37°C. Formation of dark blue colour halos around the well confirmed the presence of biosurfactant.

### 2.6. Determination of Biosurfactant Producing Endophytic Fungi for Plant Growth-Promoting Properties

#### 2.6.1. Indole Acetic Acid (IAA) Production

IAA production by isolated endophytic fungi was assessed using the method of Gordon and Weber [29]. The culture was grown in Czapek-Dox broth amended with 50 *μ*g/ml tryptophan, followed by incubation at 28 ± 2°C for 5 to 6 days. The cultures were then centrifuged at 10,000 rpm for 15 minutes and 3 ml of supernatant was taken from culture and 2-3 drops of O-phosphoric acid were added. Further, 4 ml of Salkowski reagent (1 ml of 0.5 M FeCl_3_ in 50 ml of 35% HClO_4_) was added to each aliquot. Samples were then incubated for 25 minutes at room temperature. The observance was read at 530 nm. The obtained auxin quantification values were recorded by preparing calibration curve made using IAA as standard (10–100 *μ*g/ml).

### 2.7. Extraction and Detection of IAA from Fermentation Broth by High Performance Thin Layer Chromatography (HPTLC)

Extraction of IAA was carried out from 10-day-old fermented broth of isolated endophytic fungi. Culture supernatant was acidified to pH 2.0 using HCl (1 N) and extracted twice with double volumes of ethyl acetate. Ethyl acetate fractions were collected and evaporated in a rotary evaporator at 40°C. Extract was dissolved in minimum volume of methanol and used for analysis. Extracted fungal IAA (2 *μ*l) was applied on silica gel coated plate (Silica gel G f254, thickness 0.25 mm, Merck®, Berlin, Germany) via LINOMAT (CAMAG, Darmstadt, Germany) and run in a solvent system of n-butanol : ammonia : water (10 : 1 : 10 v/v/v). At last, TLC plate was sprayed with Salkowski reagent to visualize IAA band. Standard IAA was also applied on to the plate to identify fungal IAA on the basis of *Rf* value.

### 2.8. Siderophore Production

Siderophore (an iron chelator) production was determined by modified chromazurol S (CAS) method developed by Schwyn and Neilands [30]. The PDA medium was supplemented with CAS (60.5 g in 50 ml), iron III solution (1 mM FeCl_3_·H_2_O), 10 mM HCl (10 ml), and hexadecyltrimethylammonium bromide (HDTMA) (72.9 mg in 40 ml). Development of yellow-orange halo zone around the fungal mycelia was considered as a positive for siderophore production. Hydroxamate siderophores was determined using the Csaky test [31] and catechol-type siderophores was estimated by Arnow's method [32].

### 2.9. Quantitative Phosphate Solubilization Activity

The ability of isolated endophytic fungi to solubilize the phosphate was determined by inoculating on Pikovskaya's agar medium (PVK) [33] containing tricalcium phosphate (TCP) (g/L) : 0.5 g (NH_4_)_2_SO_4_, 0.1 g MgSO_4_·7H_2_O, 0.02 g NaCl, 0.02 g KCl, 0.003 g FeSO_4_·7H_2_O, 0.003 g MnSO_4_·H_2_O, 5 g Ca_3_(PO_4_)_2_, 10.0 g glucose, 0.5 g yeast extract, 15.0 g agar, and 1000 ml distilled water. The plates were incubated at 28 ± 2°C for 5 to 6 days. A clear halo zone around the fungal mycelia indicates solubilization of mineral phosphate plates and no zone indicates a negative result.

Quantitative estimation was carried out in 100 ml of Pikovskaya's broth medium supplemented with 0.5% of tricalcium phosphate. Each of the isolated endophytic fungi was inoculated in to the medium and incubated in shaking condition at 120 rpm up to 10 days at 28°C. Control was also prepared without fungal inoculation. 15 ml of control and culture samples were withdrawn after 2, 4, 6, 8, 10 days of incubation. Culture was centrifuged at 5000 rpm for 15 min, clear supernatant was collected and filtered through syringe filter, and pH was recorded with a digital pH meter. Amount of soluble phosphorus in the cell free culture supernatant was determined using phosphomolybdenum blue method [34] by taking absorbance at 820 nm and compared with the standard curve of KH_2_PO_4_.

### 2.10. Hydrogen Cyanide (HCN) Production

HCN production was evaluated by streaking fungi on King's B agar medium supplemented with glycine. Whatman No. 1 filter paper soaked in picric acid (0.05% solution in 2% sodium carbonate) was placed in the lid of each Petri plate. The plates were then sealed airtight with Parafilm and incubated at 30°C for 48 h. A colour change of the filter paper from deep yellow to reddish-brown colour was considered as an indication of HCN production.

### 2.11. Antagonistic Potential against Plant Pathogens

Antagonistic activity of isolated endophytic fungi was evaluated against phytopathogenic fungi* Fusarium oxysporum and Aspergillus niger* by direct opposition method [35]. Mycelia of pathogenic fungi were placed at about 1 cm apart from the wall of PDA plate and, at the opposite, test isolate was placed. Control plates were also prepared without inoculation of test isolates. After 2 weeks of incubation at 25 ± 2°C, the plates were evaluated for evidence of fungal growth inhibition as compared with control [36]. Zone of inhibition was determined as described by the Riungu et al. [37]:

(2) $$\begin{array}{c} \%\text{ Inhibition of mycelia growth}=\frac{A-B}{A}\times 100, \end{array}$$

where *A* is mycelial growth of pathogen in absence of antagonists; *B* is mycelial growth of pathogen in presence of antagonists.

### 2.12. In Vivo Plant Growth Promotion Assay

Chilli seeds obtained from the local market were surface sterilized by 0.1% HgCl_2_ for 5 minutes and then 70% alcohol for 2 minutes followed by rinsing with sterile D/W five times.* X. regalis* was grown in PDA broth for 48 hours at 25 ± 2°C with continuous shaking at 140 rpm. Surface sterilized seeds of chilli were inoculated by soaking in the culture suspension of* X. regalis* for 30 minutes and air-drying them. The seeds were then transferred on a double-layer moistened filter paper in Petri dish up to one week. For the maintenance of sufficient amount of moisture for germination, 5 ml of distilled water was added every other day in Petri dish and incubated at 30°C in a light condition. After 15 days, germinated chilli seedlings were planted separately in plastic bags filled with sterile garden soil. The following greenhouse treatments included C, untreated chilli seedlings as control, and X1,* X. regalis* inoculum treated chilli seedlings. All plants were watered twice daily. For each treatment, the plants of all the bags were harvested 3 weeks after the emergence of seedlings and washed and morphological characteristics like root length, shoot length, and dry and wet weight of stem and root of each plant were measured.

### 2.13. Effect of* X. regalis* Seed Treatment on Chlorophyll (Chl), Nitrogen (N), and Phosphorus (P) Content in Chilli Plants

In order to determine the capability of* X. regalis* to enhance growth in plants, surface sterilized seeds of chilli were treated with their culture as described above. Estimation of chlorophyll a, b and total chlorophyll from control and treated plants was carried out by using the method of Lichtenthaler and Wellburn, 1983 [38]. 0.5 g of leaf sample was homogenized with 5 ml of 80% prechilled acetone. The extract was centrifuged at 5000 rpm for 5 minutes. Supernatant was made up to 100 ml with 80% acetone and the absorbance was taken at 663 and 646 nm. Chlorophyll content (mg/g) of fresh weight was calculated using following formula:

(3) Chl a=12.25×A663−2.79×A646Chl b=21.5×A646−5.1×A663Total  Chlorophylls=Chl a+Chl b.

For the determination of nitrogen and phosphorus content, plants were uprooted gently without disturbing the root system and soil particles attached with roots were removed by washing gently under running tap water. Whole plants from control and treatment were dried in an oven at 70°C for 48 h and then powdered for N and P determinations. N content from the plants was estimated by Microkjeldahl method [39] and P content by vanadomolybdate method [40].

## 3. Results

### 3.1. Isolation and Identification of Endophytic Fungi

On the basis of morphological as well as ITS gene sequence analysis, a fungal strain named as* Xylaria regalis* belongs to the genus* Xylaria* was isolated from the cones of* Thuja plicata* (Donn ex. D. Don.) (Supplementary Figure 1). After successful identification, nucleotide sequence was deposited to NCBI with accession number MG451051.

### 3.2. Phylogenetic Analysis

**Figure 1:**
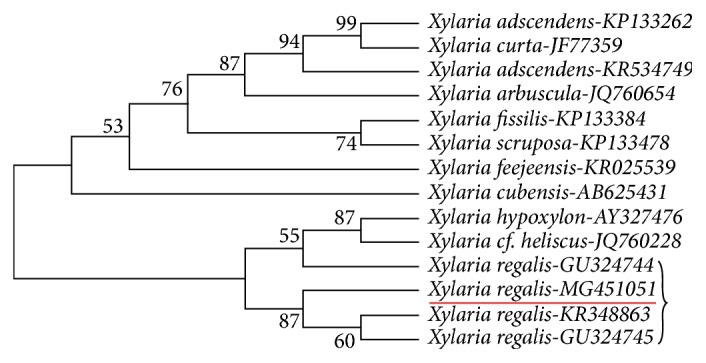
Phylogenetic analysis of ITS gene sequence showing the place of isolated endophytic* X. regalis* and its relationship among other species of the* Xylaria *genus (accession numbers are provided).

In our phylogenetic analysis, we used taxa of* Xylaria*, for which ITS gene sequences were available in GenBank. The evolutionary history was inferred using the maximum parsimony method. The most parsimonious tree is shown in Figure 1. The percentage of replicate trees in which the associated taxa clustered together in the bootstrap test are shown above the branches. These results confirm that the isolated endophytic fungi species unequivocally belongs to the genus* Xylaria* (Figure 1).

### 3.3. Screening of Biosurfactant Production

**Figure 2:**
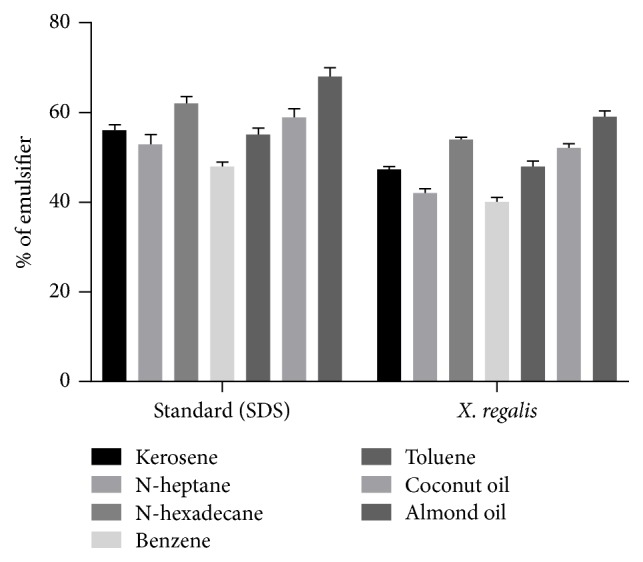
Emulsification index test of* X. regalis* against various aliphatic and aromatic compounds and different oils.

The potential of biosurfactants production by isolated endophytic fungi* X. regalis* was screened by various methods like emulsification capacity assay, oil spreading assay, drop collapse assay, and blue agar plate method. In emulsification assay,* X. regalis* was firstly screened with kerosene. Emulsification index of 30% or more was considered as positive emulsification activity.* X. regalis* showed more than 40% emulsification index (Supplementary Figure 1). It was additionally screened with aliphatic hydrocarbon (n-heptane and n-hexadecane), aromatic hydrocarbon (benzene and toluene), and different oils (coconut and almonds) for their emulsification capacity in comparison to SDS as standard.* X. regalis* showed higher emulsification index against almond oil (Figure 2).

**Figure 3:**
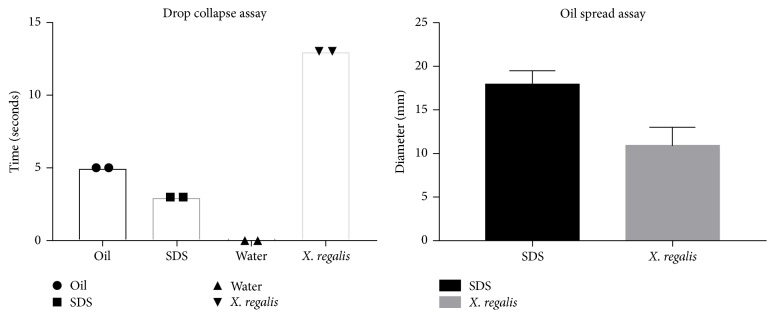
Bar graph representing results of drop collapse assay and oil spread assay.

However, it was able to maintain its emulsification activity for more than one week. Following the results of emulsification assay, drop collapse assay was performed. This assay relies on the destabilization of liquid droplet by surfactants. Consequently, drops of a cell suspension or culture supernatant of* X. regalis* were placed on oil. In the event, if the liquid does not contain surfactants, the polar water particles are repulsed from the hydrophobic surface and the drops remain stable. On the other hand, if the liquid contains any kind of surfactant, drop will collapse due to the force or interfacial tension between the liquid and the hydrophobic surface (Supplementary Figure 1). Surfactant concentration and correlation between the surface and interfacial tension have effect on the stability of drop. In comparison with standard (SDS and oil),* X. regalis* took 13 seconds to collapse (Figure 3).

Oil spreading assay is a furthermore authenticating step for confirmation of drop collapse assay results. In oil spreading assay, concentration of biosurfactants is directly proportional to the area of oil displacement (Supplementary Figure 1). Oil spread assay was performed with regard to time and diameter.* X. regalis* showed positive result for oil spread assay (Figure 3). BAP method is another semi-quantitative technique for detection of biosurfactant in which CTAB and methylene blue are used.* X. regalis* was grown on the plate and produced dark blue halos (Supplementary Figure 1).

### 3.4. Depicting Plant Growth-Promoting Traits of* X. regalis*

#### 3.4.1. Detection of IAA Production and Phosphate Solubilization

**Figure 4:**
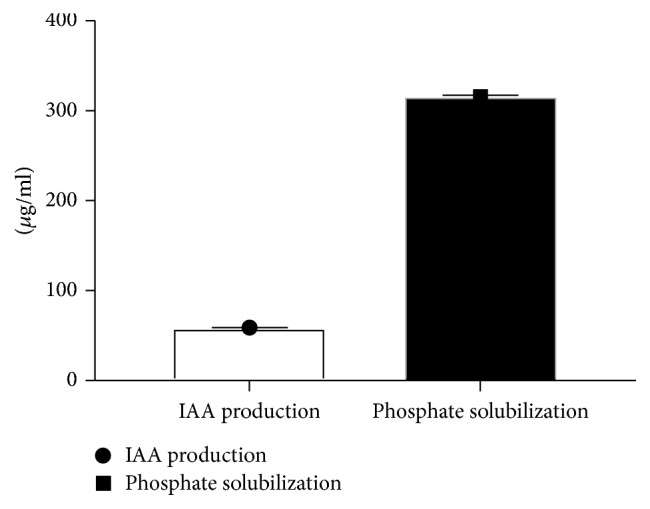
Quantitative estimation of IAA and phosphate solubilization of* X. regalis*.

*X. regalis* was able to produce IAA in test media. Extent of IAA produced by* X. regalis* (58 *μ*g/ml) is seen in Figure 4. Furthermore, HPTLC chromatogram of ethyl acetate extract exhibited clear red colour band of IAA with *Rf* value 0.35 corresponding to standard IAA when sprayed with Salkowski's reagent (Supplementary Figure 2). Phosphate solubilization ability of* X. regalis* was determined on PVK agar medium supplemented with TCP. It was identified as potential phosphate solubilizing as it was able to solubilize inorganic phosphate and formed a clear zone around the colony (Supplementary Figure 2). Quantitative assessment revealed* X. regalis* as a strong phosphate solubilizer (316 *μ*g/ml) (Figure 4).

### 3.5. Detection of Siderophore and HCN Production

To determine if* X. regalis* was producing a siderophore under iron-limiting conditions, the CAS assay was used. Formation of an orange halo around the growth into the CAS plate indicates that siderophore is produced by the fungi.* X. regalis* produces a siderophore under iron-limiting conditions, which is not produced when sufficient iron is available (Supplementary Figure 2). However, once it had been indicated that* X. regalis* produced a siderophore, the chemical type of the siderophore was determined by Arnow's assay and iron-perchlorate assay. In Arnow's assay, formation of red colour indicates the production of catechol-type siderophore and in iron-perchlorate assay; formation of an orange-red colour is indicative of a hydroxamate-type siderophore.* X. regalis* was found to produce hydroxamate-type siderophore (Supplementary Figure 2).

*X. regalis* was also positive for HCN production as indicated by a very deep red colour on the filter paper (Supplementary Figure 2).

### 3.6. Antagonistic Potential against Plant Pathogens

**Figure 5:**
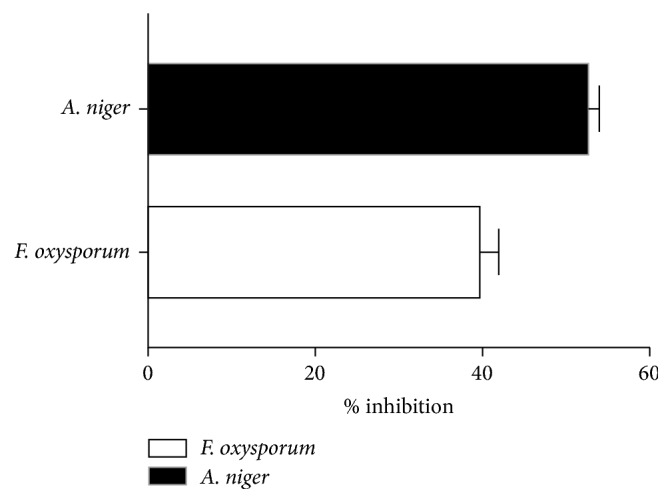
Antagonistic activity of* X. regalis* against* F. oxysporum* and* A. niger*.

Antagonistic activity of* X. regalis* was tested against* F. oxysporum* and* A. niger*.* X. regalis* showed significant inhibitory activity against both important plant pathogens. Antagonistic activity against* A. niger* was higher than the* F. oxysporum*. Results of antagonistic activity are represented in (Figure 5).

### 3.7. In Vivo Plant Growth Activity of Chilli Seedlings

**Figure 6:**
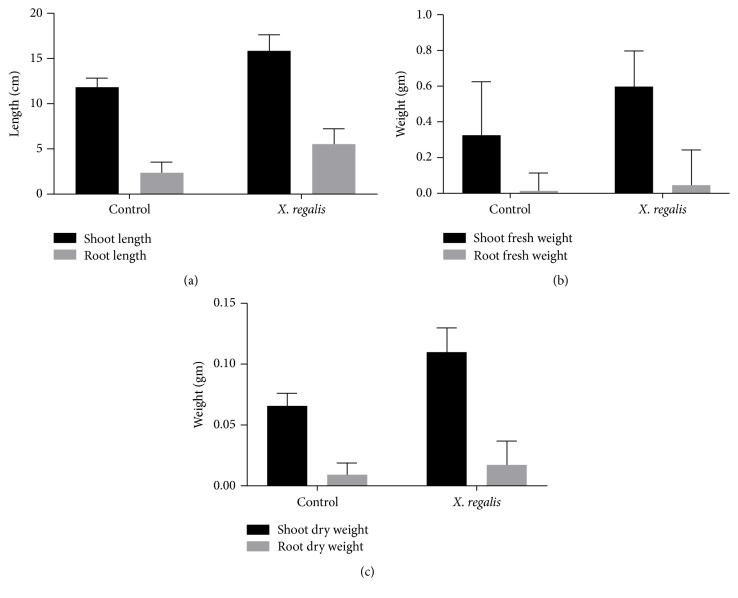
Effect of* X. regalis* inoculation on (a) shoot and root length; (b) shoot and root fresh weight; (c) shoot and root dry weight.

**Figure 7:**
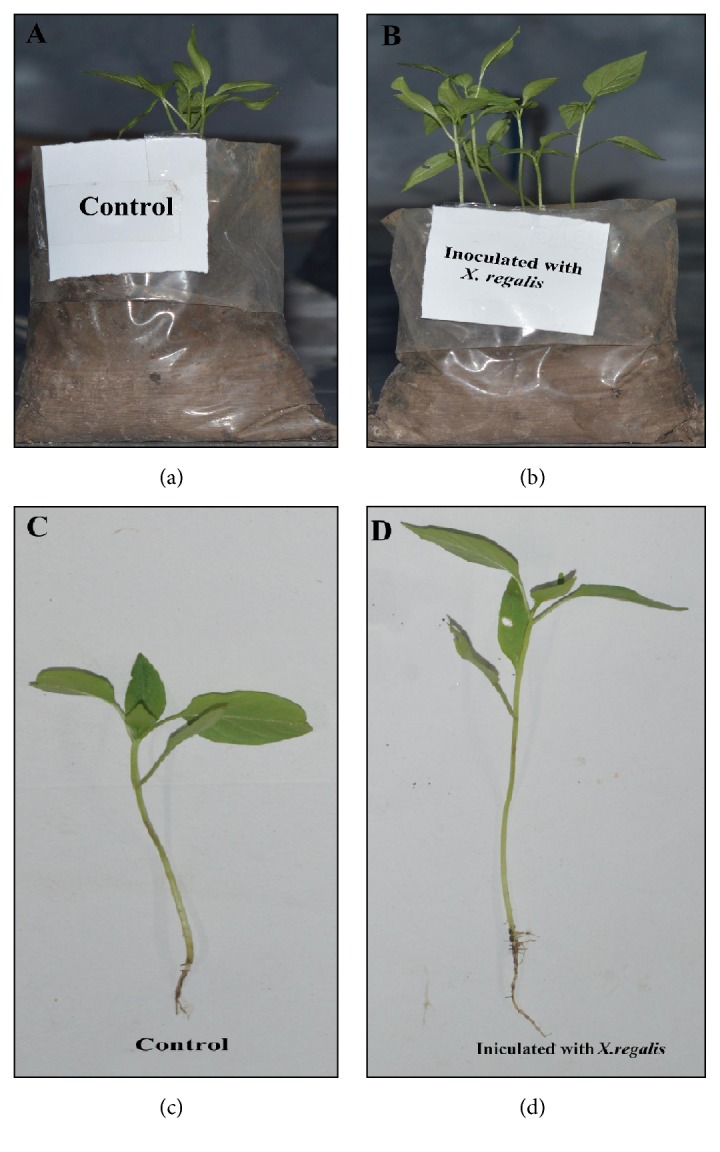
Effect of* X. regalis* inoculation on plant growth of chilli seedling: (a) control; (b) inoculated with* X. regalis*; (c) root and shoot length of control plant; (d) shoot and root length of* X. regalis* inoculated chilli seedling.

Endophytic fungi* X. regalis* was significantly found in enhancing the length of chilli seedlings and facilitating the plant growth. Results reveal that, shoot length (15.8 cm/plant) and root length (5.5 cm/plant) are statistically significant in treated plants over uninoculated control (Figure 6(a)). A significant increase in shoot dry matter of chilli seedlings was observed in response to endophytic fungal isolate* X. regalis*. A single inoculation of* X. regalis* was recorded with significant increase in root dry weight (0.017 g/plant) (Figures 6(b) and 6(c)). Overall results showed that the inoculation of* X. regalis* significantly increases the shoot length, root length, and dry matter production of shoot and root of chilli seedlings (Figure 7).

### 3.8. Chlorophyll, Nitrogen, and Phosphorus Concentration in Chilli Plants

**Figure 8:**
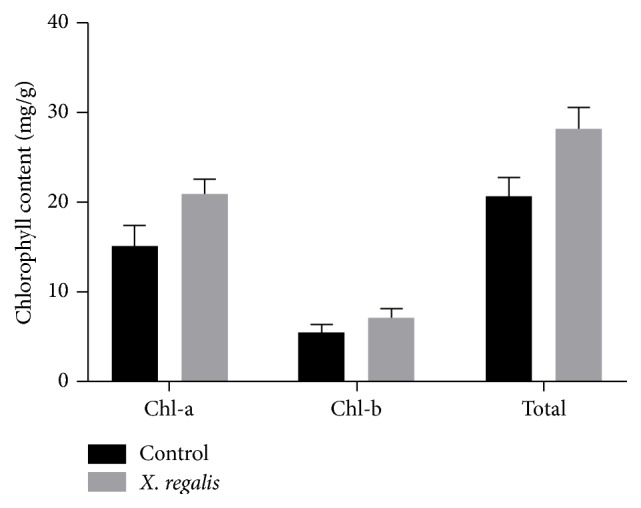
Effect of* X. regalis* inoculation on chlorophyll content (mg/g) in chilli plant.

**Figure 9:**
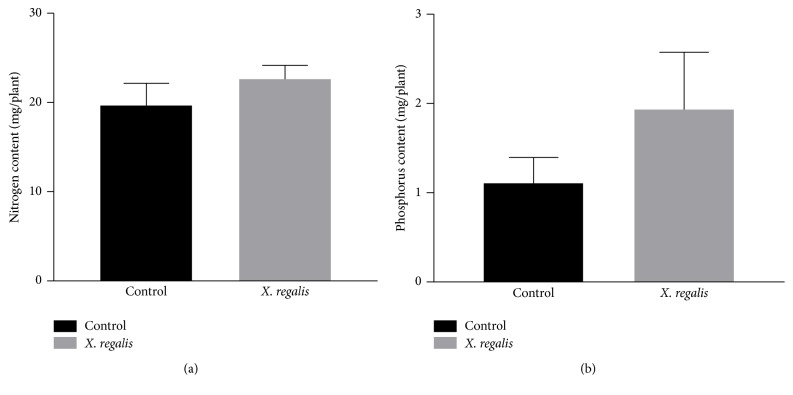
Effect of* X. regalis* inoculation on (a) N content (mg/plant) in chilli plant and (b) P content in chilli plant (mg/plant).

*X. regalis* significantly improves the chlorophyll content, and obtained values are calculated as 28.20 mg/g in chilli plant over the control 20.64 mg/g (Figure 8). Similarly, N and P content in plants were also found to be significantly increased after treatment with* X. regalis,* when compared with control plants. Total N content in treated plant is 22.66 mg/plant over the control 19.74 mg/g, while P content in treated plant is 2.93 mg/plant over the control 1.10 mg/plant (Figures 9(a) and 9(b)).

## 4. Discussion

In recent times, environmental issues are rising due to direct or indirect use of various chemical agents like pesticides, fertilizers, and so on in agriculture and horticulture field for crop production. Therefore, there is an urgent need for attractive and alternative environment friendly biological agent for the betterment of plant growth and to control plant pathogens, which can replace synthetic harmful chemicals in future. Endophytic fungi are of special interest, as they have many properties, which enhance the growth of plants. Recently, interest in biosurfactants has increased because of their diversity, flexibility in operation, and more ecofriendly than chemical surfactants with potential for a wide range of applications including the control of plant and human pathogens [41–44]. Biosurfactants are low molecular weight, amphiphilic, surface active compounds produced by microorganisms (bacteria, yeast, and fungi). Large numbers of biosurfactant producing bacteria are isolated from the rhizosphere of the plant which enhances the plant growth promotion [45, 46]. Therefore, endophytic fungi might be able to produce different kinds of biosurfactant molecules which are involved in direct or indirect plant growth promotions. However, the whole concept is novel with researches in initial stage as well as very few reports regarding the production of biosurfactants by endophytic fungi is known [47, 48].

To our knowledge, this study is the first report concerning the isolation of endophytic fungi which can produce biosurfactants and characterization of its plant growth-promoting potential. In the present study, endophytic fungal strain named as* X. regalis* belongs to the genus* Xylaria* was isolated from the cones of* Thuja plicata* (Donn ex. D. Don.).* X. regalis* was screened for its biosurfactants production ability and to assess their role as potential plant growth promotion.

Biosurfactant production ability of* X. regalis* was screened by various methods like emulsification assay, drop collapsing assay, oil displacement test, and CTAB agar plate method. During the screening of biosurfactant production, emulsification activity is one of the most important methods which determine the productivity of bioemulsifier [49], given as a percentage of the emulsified layer divided by the total height of the liquid column. Emulsification activity was measured by the E24 index in which* X. regalis* showed good emulsification index with all the hydrocarbons tested, which includes kerosene, n-hexane, n-heptane, benzene, toluene, coconut oil, and almond oil. Drop collapse and oil displacement methods are sensitive and relatively easy to perform, as they require a small quantity volume of sample and do not require specialized equipment. Drop collapse assay is based on the destabilization of liquid droplets by surfactants.* X. regalis* was positive towards the drop collapse and oil spread assay. Blue agar plate (CTAB agar) method was developed by Siegmund and Wanger [50] and used for the detection of extracellular glycolipids.* X. regalis* also had shown positive results in blue agar plate method. From the above of all methods, it was confirmed that* X. regalis* is a potent biosurfactant producing endophytic fungi.

Furthermore, our results demonstrated that isolated endophytic fungi* X. regalis* promote plant growth through production of different plant growth regulators like IAA, siderophore production, phosphate solubilization, HCN production, and antagonistic activity against common agriculture phytopathogens like* F. oxysporum* and* A. niger.* During the plant growth-promoting experiments,* X. regalis* was found significantly to produce IAA.* X. regalis* produce IAA inside the plant tissue which might have played an important role in host plant development and growth. IAA production ability of* X. regalis* indicated that it can enhance root and shoot development when inoculated with the chilli seedlings.

Siderophore production is one of the important activities for plant growth promotion in which microbes chelate iron (Fe_3_^+^) by producing different types of siderophore molecules (strongest Fe_3_^+^ binding agents) making iron unavailable to the phytopathogens in to the rhizosphere [51]. However, production of siderophore is an important factor for phytopathogen antagonism and developing growth of the plant [52].* X. regalis* was found to produce hydroxamate-type siderophore, which can inhibit phytopathogen by competing for iron in rhizosphere soils.

Phosphorus is also a major nutrient which plays an important role in the improvement of plant growth. It is present in the soil in form of insoluble phosphates. We found that* X. regalis* have the ability to convert insoluble phosphate into soluble form by producing low molecular weight organic acids [53]. Moreover,* X. regalis* was also able to produce HCN which plays an important role in diseases suppression [54]. Along with various plants growth-promoting activities of endophytic biosurfactant producing fungi* X. regalis* also showed a significant antagonistic activity against phytopathogens* F. oxysporum* and* A. niger*.* X. regalis* produces a variety of antifungal and active metabolites which shows a potent antagonistic activity against phytopathogens along with plant growth-promoting traits, which can be explored as novel source of biocontrol agents.

Treatment of chilli seeds with biosurfactant producing endophytic fungi* X. regalis* significantly improved seedlings in germination and growth. The inoculation of* X. regalis* increased shoot length, root length, dry matter production of shoot and root, chlorophyll, and N and P of chilli seedlings as compared to control. For the growth and productivity of plants, N and P are vital nutrients. Both are helpful for increasing plant biomass and can also serve as a triggering factor for the virulence of opportunistic plant pathogens [55]. Therefore, results of the present study demonstrate that* X. regalis* inoculation can increase nutrient availability in plants and overall plant growth.

Many reports have shown that endophytic fungi can support plant growth promotions with efficient biocontrol activity [56–59]. Various mechanisms have been suggested on this activity of plant growth promotion like reducing the incidence of seed mycoflora [60], increasing of amylase activity [61], and production of phytohormones such as IAA [62]. This reveals its ability to supply sustainable options for agriculture to improve the growth of crop plants.

## 5. Conclusion

In conclusion, this study provided results for the first time with activity of biosurfactant producing endophytic fungi* X. regalis* inside the plant tissue of* Thuja plicata* (Donn ex. D. Don.). Biosurfactant production by endophytic fungi* X. regalis* which can enhance plant growth is a positive indication for its potent role in sustainable agriculture. Biosurfactants can be widely used in areas of agriculture for direct or indirect plant growth promotions as they have biocontrol activity and to improve the quality of agriculture soil via biodegradation of pollutants. However, the exact mechanism of surfactant in assisting other system as antagonistic agent is not studied in detail which warrants investigations. Up to date, only* Pseudomonas* and* Bacillus* appear in literature as producers of biosurfactants. Further field experiments will greatly reveal the applicability of this potent biosurfactant producing endophytic fungi for alleviating environmental stress and thereby increasing plant productivity. Therefore, our study will help in the discovery of novel biosurfactants as well as their ecofriendly use in agriculture field.

## Supplementary Materials

Supplementary Materials Supplementary Figure 1: (A) purified endophytic fungi on PDA; (B) amplification of DNA by PCR; (C) emulsification index test; (D) blue agar assay; (E) oil spreading assay; (F) drop collapse assay. Supplementary Figure 2: (A) HPTLC analysis of extracted IAA from* X. regalis*; (B) phosphate solubilization; (C) siderophore production; (D) hydroxamate-type siderophore production; (F) HCN production.
