# Supplementary material for: Physiological and Molecular Characterization of Biosurfactant Producing Endophytic Fungi Xylaria regalis from the Cones of Thuja plicata as a Potent Plant Growth Promoter with Its Potential Application
Source: Biomed Res Int. 2018 May 13;2018:7362148. doi: 10.1155/2018/7362148 (PMC5971342; doi:10.1155/2018/7362148)
Supplement: Supplementary Materials — Supplementary Figure 1: (A) purified endophytic fungi on PDA; (B) amplification of DNA by PCR; (C) emulsification index test; (D) blue agar assay; (E) oil spreading assay; (F) drop collapse assay. Supplementary Figure 2: (A) HPTLC analysis of extracted IAA from X. regalis; (B) phosphate solubilization; (C) siderophore production; (D) hydroxamate-type siderophore production; (F) HCN production. [file 7362148.f1.pdf]

Supplementary Figures

Supplementary Figure 1

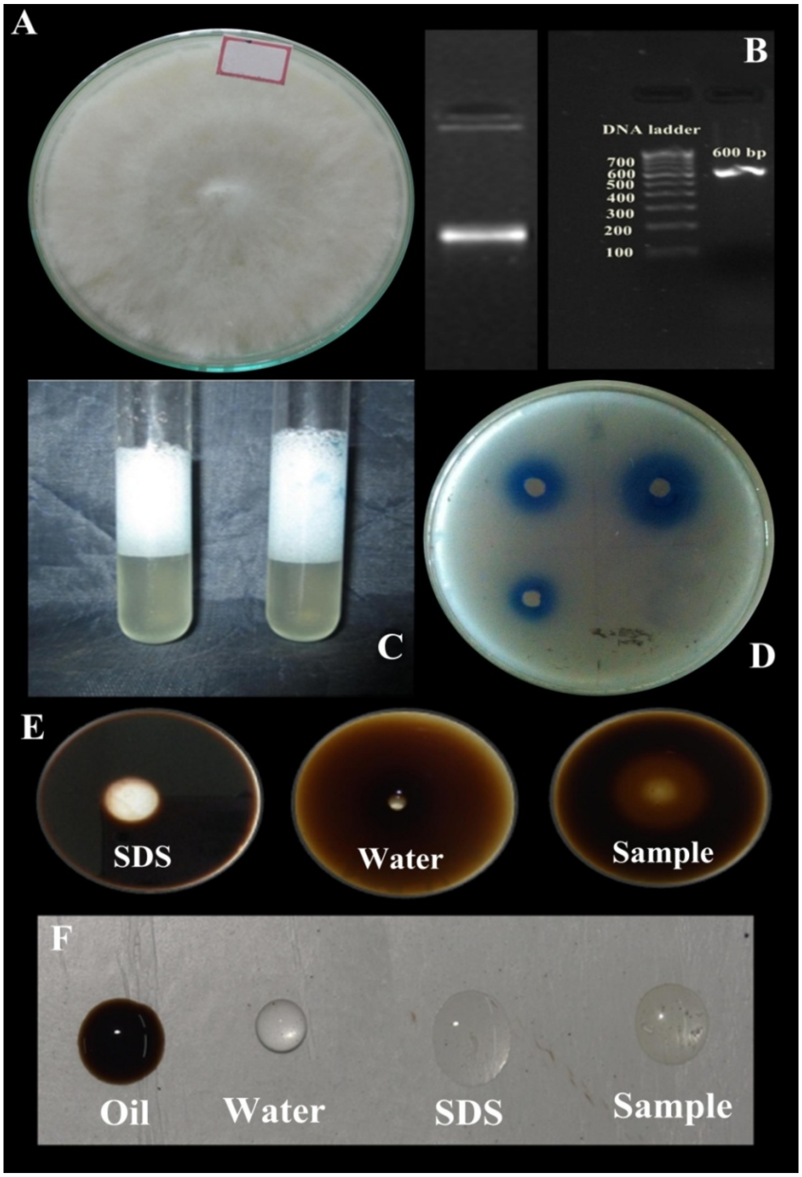

Supplementary Figure 1: **A) Purified endophytic fungi on PDA B) Isolation and amplification of DNA by PCR C) Emulsification index test D) Blue agar assay E) Oil spreading assay F) Drop collapse assay**

Supplementary Figure 2

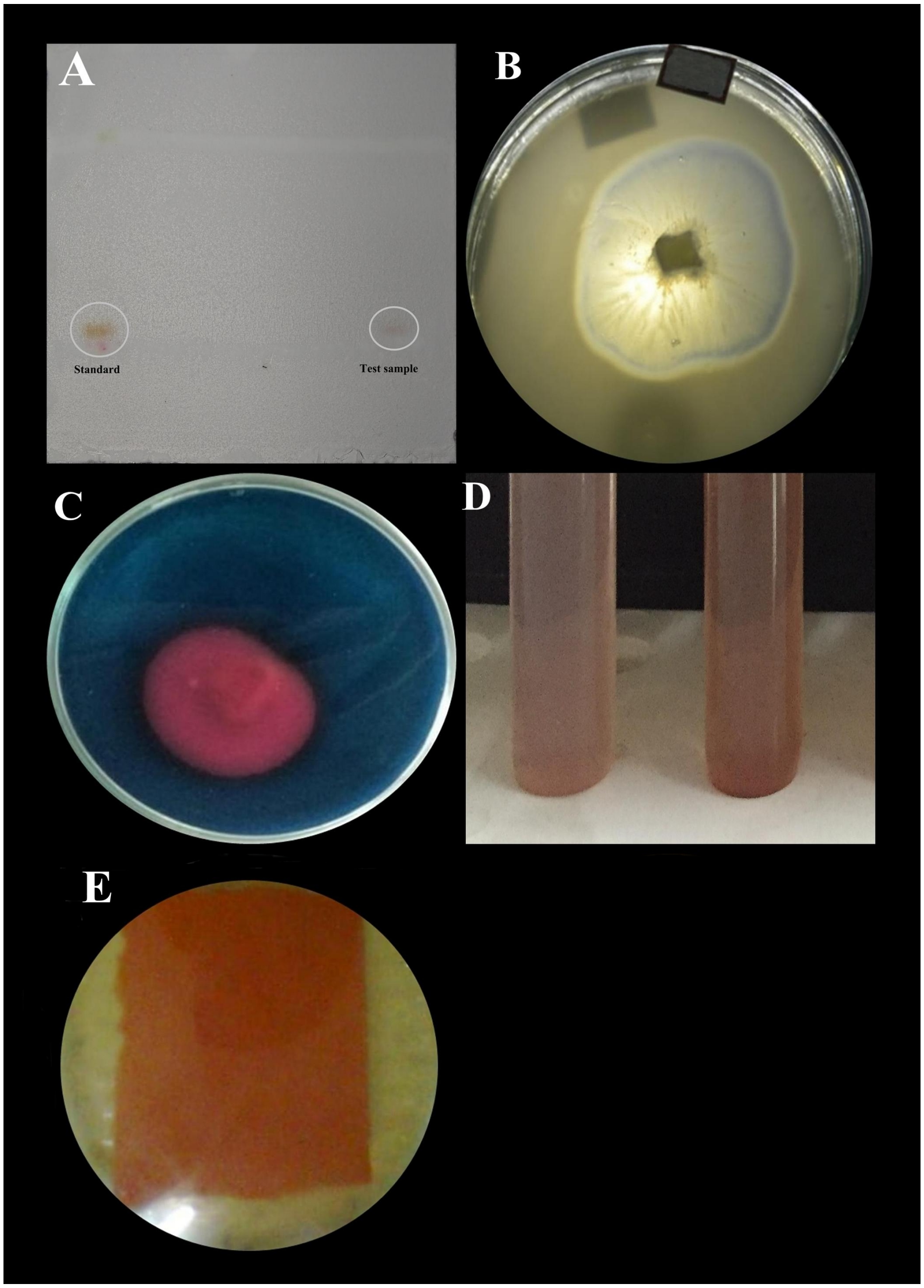

<u>Supplementary Figure 2:</u> A) HPTLC analysis of extracted IAA from *X. regalis* B) Phosphate solubilization C) Siderophore production D) Hydroxamate type siderophore production E) HCN production.
